# Outcomes of open, laparoscopic, and hand-assisted laparoscopic surgeries in elderly patients with right colon cancers: A case–control study

**DOI:** 10.1097/MD.0000000000011907

**Published:** 2018-08-21

**Authors:** Mingtian Wei, Xubing Zhang, Pingfan Ma, Wanbin He, Liang Bi, Ziqiang Wang

**Affiliations:** a Department of Gastrointestinal Surgery, West China Hospital, Sichuan University; b State Key Laboratory of Biotherapy and Cancer Center, West China Hospital, Sichuan University and Collaborative Innovation Center, Chengdu; c The People's Hospital of Leshan, Leshan, China.

**Keywords:** elderly patients, hand-assisted laparoscopic surgery, laparoscopic surgery, outcomes, right colon cancer

## Abstract

An increasing proportion of patients aged more than 70 years old are suffering from colorectal cancers. This study aimed to compare the short- and long-terms outcomes between open surgery (OS) or conventional laparoscopic surgery (LS) and hand-assisted laparoscopic surgery (HALS) in treatment of these elderly patients with right colon cancers.

We retrospectively reviewed patients who underwent right colon resections for cancers in our institution between June, 2009 and December, 2014. Short- and long-terms outcomes including surgical endpoints, postsurgical recovery data, postoperative morbidity and mortality, overall survival and disease-free survival were compared among OS, LS, and HALS groups. All data were analyzed by SPSS 22.0.

Finally, 69 consecutive patients (OS = 26, LS = 24, HALS = 19) with right colon cancers were included in the analysis. Compared with OS, HALS was associated with less time to first anus exhaust (*P = *.013), first liquid diet (*P = *.045), and first soft diet (*P = *.036). Meanwhile, there were significant less operative time (*P = *.0027), blood loss (*P < *.001), and less time to first liquid diet (*P = *.009) in HALS, compared with LS. In regards to long-term outcomes, there were no significant differences in overall survival and disease-free survival among the 3 groups.

Compared with OS or LS, HALS may be more favorable in the treatment of elderly right colon cancers with decreased surgical time and postoperative recovery, and comparable cancer-specific survivals.

## 1 Introduction

Colorectal cancer (CRC) accounts for the second most common cancer in Western countries, with more than 70% of patients aged 65 years or over.^[1]^ In China, existing evidences have revealed an increasing proportion of patients aged 70 years or over suffer from CRC.^[2,3]^ Although the elderly patients are deemed as high-risk surgical candidates with probable increased perioperative morbidity and declined physical function; however, modern minimally invasive surgical improvements and people's longer life expectancy have universalized surgical resection of CRC among elderly patients. Up to now, in the specific patients’ population of aged 70 years or older, a considerable amount of literatures have observed favorable postoperative morbidity and fast recovery in laparoscopic colorectal resection, compared with open surgery.^[4–8]^

At the same time, recent years have witnessed a considerable progress of minimally invasive surgery in the management of CRC, particularly in the usage of laparoscopy-assisted technique.^[9,10]^ As a theoretic superiority to restore the spatial orientation, hand-assisted laparoscopic surgery (HALS) has been verified equivalent hospitalization time, postoperative morbidity, and controversial operative time over LS in the whole population of right colon cancers.^[11,12]^ However, little has been investigated on the comparison between OS or LS and HALS in a specific population of elderly patients with right colon cancers.

To address this notion, we performed this present study to compare the short-term and long-term outcomes between OS or LS and HALS in the elderly patients suffering right colon cancers.

## 2 Materials and methods

### 2.1 Study design

All consecutive patients undergoing resection of right colon cancer were eligible in a retrospectively colorectal cancers database. The patients were from Department of Gastrointestinal Surgery West China Hospital and operated by one experienced surgeon (author ZW) between June 2009 and December 2014. All patients signed informed consent agreement based on their own decision on the surgical procedure.

### 2.2 Inclusion and exclusion criteria

Patients fulfilling the criteria were included in the analysis: confirmed diagnosis of right colon cancer; age of diagnosis was 70 years or older; surgical procedure was open, conventionally laparoscopic, or hand-assisted laparoscopic; elective operation with a radical resection (R0 resection). Exclusion criteria were distant metastasis, multiple cancers, psychological disorder, conversion to open surgery, and emergency surgery due to obstruction or perforation. All patients had to be able to tolerate surgery during general anesthesia. Flexible colonoscopy and biopsy, contrast-enhanced CT scans of the abdomen and chest were routinely conducted in all patients. Patients diagnosed with stage II or higher were candidates for 5-fluorouracil-based adjuvant chemotherapy. Oral adjuvant chemotherapy was considered for those patients who would not tolerate intravenous chemotherapy.

### 2.3 Surgical procedures

All operations were performed by the same experienced laparoscopic team. Our latest publication describes the modification of HALS procedure.^[13]^ Briefly, after establishing pneumoperitoneum, the patient was placed in the supine position with legs spread apart. The surgeon stood between the patient's legs while 2 assistants stood on the left side of the patient. A 5- to 7-cm midline incision was made around the umbilicus to allow placement of the Lap-Disc device (Ethicon). Three trocars were placed: one in the left lower quadrant as the main working port, one in the upper left quadrant as the camera port, and the third one below the xiphoid used mainly for retracting the mesocolon or stomach. Firstly, the abdomen was explored in order to ascertain the resectability of the tumor and whether regional or distal metastasis was present. And then the greater omentum, transverse colon, and ileum were brought out and divided under direct sight extracorporeally. By holding the stump of the distal superior mesenteric vessels and stretching the latter, the surgeon could easily observe the superior mesenteric vein (SMV) and superior mesenteric artery (SMA). Dissection around and along the axis of the SMV was usually performed to the level of the ileocolic vein extracorporeally. After dissection around and along the axis of the superior mesenteric vein, the bowel was returned into the abdominal cavity, and the surgeon's left hand was inserted into the abdominal cavity through the Lap-Disc device to re-establish pneumoperitoneum. Lymphadenectomy and mobilization of the colon were performed intracorporeally according to the standard approach of combining European CME and Japanese D3 lymphadenectomy. After completing the lymph node dissection along the SMV, the greater omentum and mesentery of the colon were moved by the surgeon's hand, similar to the method used in the conventional laparoscopic procedure. Finally, the mobilized colon was removed through the hand-port incision, and side-to-side anastomosis was performed extracorporeally. The mesenteric defect was usually closed through the hand-port incision.

For LS, colon mobilization and vascular division were performed intracorporeally similar to HALS. The ileo-transverse anastomosis was made extracorporeally.

### 2.4 Assessment parameters

For each included patient, we obtained the baseline data: age, gender, body mass index (BMI), American Society of Anesthesiologists (ASA) class, comorbidity, preoperative blood examination, and history of gastrointestinal tumors. Surgical parameters (operation time, blood loss, and incision length), postoperative oncologic outcomes (TNM stages, differentiation, tumor size, retrieved lymph nodes, positive lymph nodes, lymphatic or vascular invasion, perineural invasion, and short-term outcomes including complications, 30-day mortality, and postoperative recovery were also recorded. All the time of postoperative recovery such as liquid time was recorded from the finish of surgery. Long-term endpoints were overall survival and disease-free survival.

### 2.5 Follow-up

All patients were followed-up regularly at 3, 6, 12, 24, 36, 48, and 60 months after surgery. Regular contrast-enhanced CT scan of the abdomen and chest, blood test, and flexible colonoscopy were done per year. Biopsy or PET-CT would be done when it is necessary to diagnose recurrence of metastasis.

### 2.6 Statistical analysis

Data were stored and updated in our institutional databases. Continuous variables were expressed as mean and range, and nonparametric Mann–Whitney *U* test was induced for analysis. Categorical variables were showed as a percentage and analyzed by Chi-Square or Fisher's exact tests. As for cancer-specific outcome analysis, Kaplan–Meier survival curves were used. A *P* value of .05 or below was deemed to be significant. All statistical analysis was performed using SPSS 22.0.

## 3 Results

**Figure 1:**
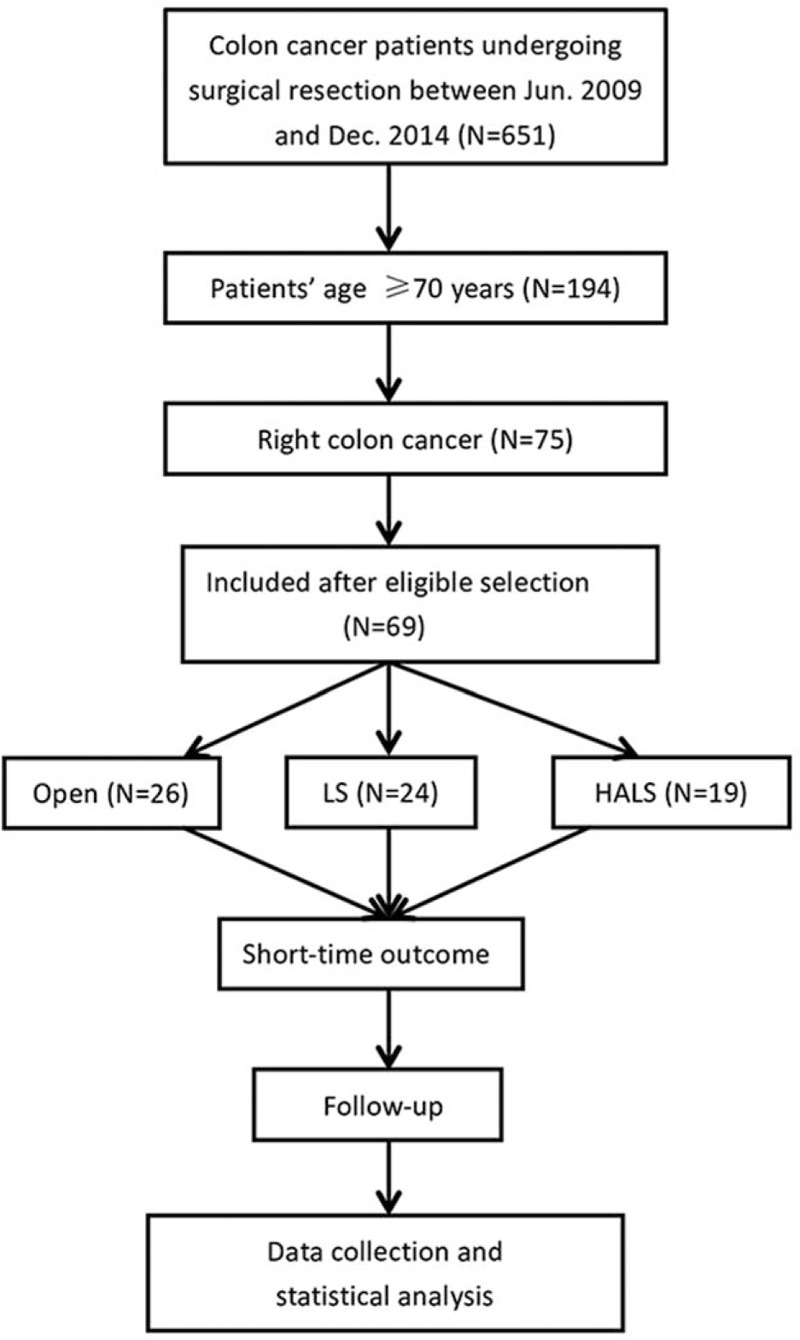
Patients’ selection diagram. HALS = hand-assisted laparoscopic surgery, LS = laparoscopic surgery.

In our databases, Between June 2009 and December 2014, 651 surgical resections with colon cancer were performed in our institution. After screening the inclusion and exclusion criteria, 69 right colon cancer patients (OS = 26, LS = 24, HALS = 19) were encountered for analysis (Fig. 1). The mean ages were 76.2 ± 0.8 years, 76.0 ± 0.7 years, and 77.1 ± 1.1 years in OS, LS, and HALS groups, respectively. There were no other major differences between groups with regard to baseline characteristics (gender, BMI, ASA score, comorbidity, and family cancer history) (Table 1).

**Table 1:**
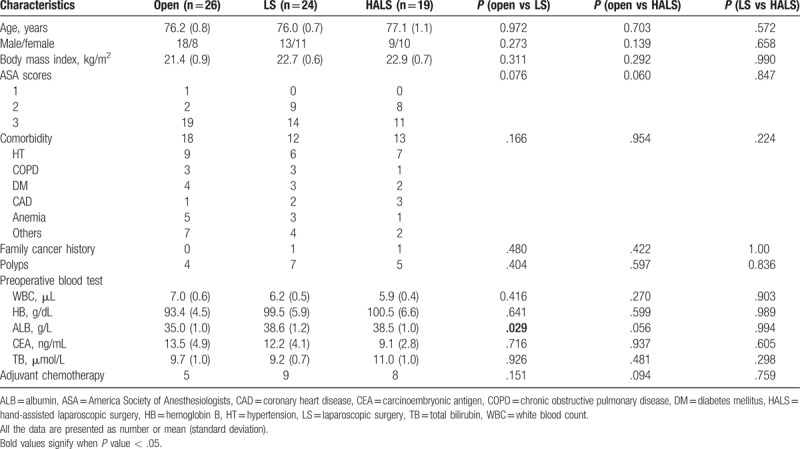
Patient demographics.

Table 2 depicted the oncological outcomes in the 3 groups. No significant differences were observed on differentiation degree, tumor maximal size, lymph node harvested, and TNM stages between groups. Both lymphatic or vascular invasion and perineural invasion were detected in either group with no statistical difference.

**Table 2:**
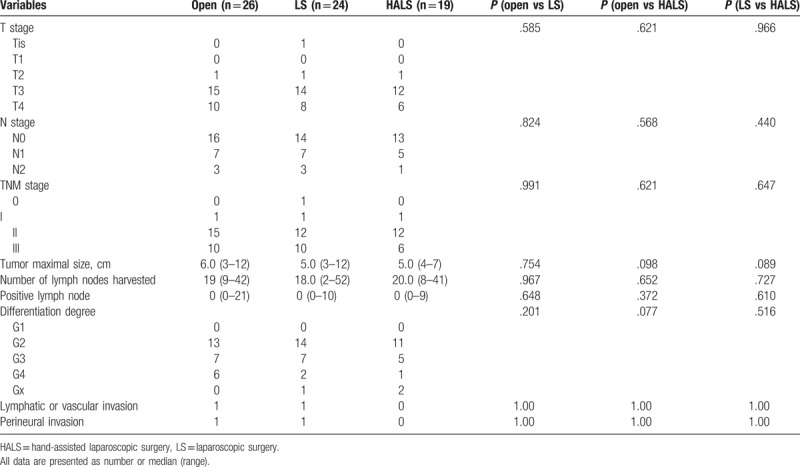
Pathological outcomes.

In terms of surgical outcomes, HALS was associated with shorter incision length (*P < *.001), shorter time to first anus exhaust (*P = *.013), gastric tube retaining (*P = *.038), less time to first liquid diet (*P = *.045), and first soft diet (*P = *.036) than OS. And the results also demonstrated that HALS had advantages in operative time (*P = *.027), blood loss (*P < *.001), and time to first liquid diet (*P = *.009) when compared with LS (Table 3). As for postoperative morbidity, there were no 30-day deaths in 3 groups. The total complication rates were similar between groups. The particular complications were listed in Table 3. One re-operation was conducted in LS group with the complication of abdominal abscess and another re-operation was performed in open group for wound dehiscence.

**Table 3:**
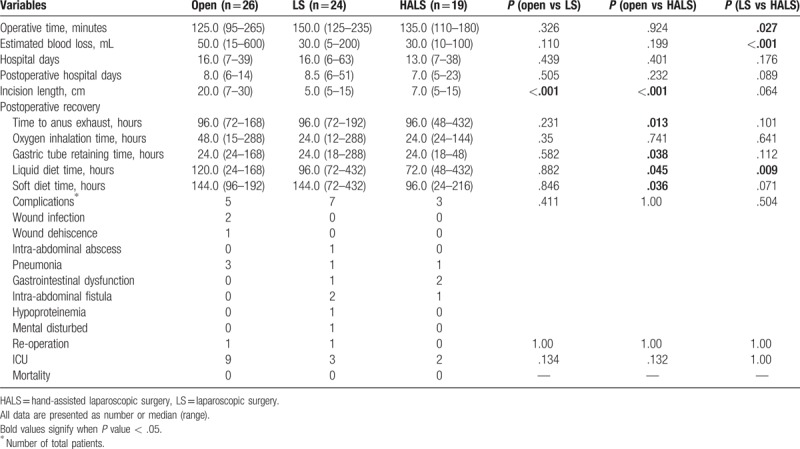
Operative and postoperative outcomes.

**Figure 2:**
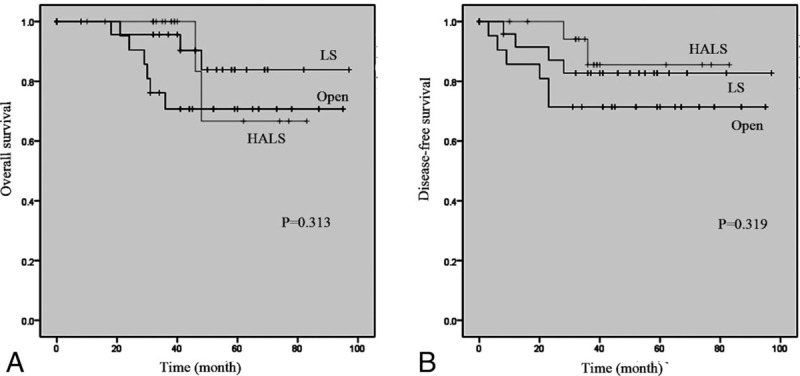
Overall survival (A) and disease-free survival (B) in open, LS, and HALS group. HALS = hand-assisted laparoscopic surgery, LS = laparoscopic surgery.

As for cancer-specific outcomes, overall survival was not significantly different between the 3 groups *(P* = .313, Fig. 2A), and the similar result was observed in terms of disease-free survival (*P* = .319, Fig. 2B). The median follow-up time was 38.5 (range 21–95), 51.5 (range 18–97), and 38.0 (range 16–83) months for OS, LS, and HALS, respectively.

## 4 Discussion

Population aging is gaining general attentions with increasing prevalence of cancers all over the world. In China, CRCs are reported to reach their peak of incidence between 61 and 70 years, and the median age has increased by 7 years in recent years.^[2,14]^ Previous well-designed studies for younger, healthier right colon cancers have revealed that laparoscopic favors improved short-term outcomes with comparable long-term cancer-specific outcomes compared to open surgery or HALS surgery.^[12,15]^ However, limited evidences show the comparison between HALS and OS or LS in treatment elderly colon cancer patients, which are deemed as high-risk surgical candidates. Thus, our present study, for the first time, summarized the short- and long-terms outcomes of elderly right colon cancer patients aged 70 years or over among OS, LS, and HALS in a retrospective case study.

As we know, advanced age is an independent risk factor for unfavorable postoperative events in cancer surgery. Indeed, the cutoff age (70 years, 75 years, or 80 years) for distinguishing elderly patients are controversial. Alves et al^[16]^ demonstrated 70 years or over as one of 4 risk factors for hospital mortality in their prospective multicenter study recruiting 1050 colorectal surgeries. Similar result was also observed in a French prospective research indicating 70 years as an independent factor for inferior short-term outcomes.^[17]^ Besides, several latest publications also defined 70 years as cutoff line in colorectal cancer surgeries.^[7,14,18]^ Accumulatively, based on the colorectal cancer prevalence in China, we considered 70 years as the cutoff age to recruit eligible right colon cancers.

In the present study, we observed significant decreased operative time in HLAS group compared with LS group. This can be explained that hand-assisted technique may facilitate the surgery procedure, which shortens the dissection time of colon, along with extracorporeal dissection of great omentum, transverse colon, and ileum in HALS. In consistent with publications in general colon cancer patients, resection of cancer in elderly patients also acquired advantage of shorter operation duration.^[19,20]^ This shortened surgical time, on the other hand, promoted postoperative recovery of those patients. Thus, it is understandable that patients in HALS group experienced shorter time to liquid diet than LS group (*P = *.009). The comparable outcomes, together with shorter operative time and liquid diet time, accumulatively, demonstrate favorable short-term outcomes in HALS technique than LS technique.

As for postoperative morbidity, several studies have presented a lower postoperative complication rate in LS surgery than open surgery in the elderly patients who undergo colorectal cancer resection.^[21–24]^ However, the comparison between HALS and LS is limited, especially rare in the subgroup of elderly right colon cancers population. Ng et al^[25]^ have demonstrated equal complications rate with 13.3% of HALS and 23% of LS in their prospective randomized controlled trial. Other data on comparison between HALS and LS in colorectal resection also omitted significant difference in the postoperative complications.^[11,19,26]^ Although performed by including the general colorectal cancer population, those reports were in agreement with our current study showing equivalent complication in groups. In particular, the total postoperative complications in the whole elderly population is 21.7%, which is similar with that in 2 publications (Guillou et al^[27]^ 32%, Pendlimari et al^[28]^ 28%) on general colorectal cancer patients. This agreement partly validates the safety of HALS in treatment of right colon cancer in the elderly.

Previous evidences have indicated considerable similar overall survival and disease-free survival between HALS and LS in the whole right colon cancer population, with the rates ranging from 80.0% to 87.3% and from 75.2% to 81.8%, respectively.^[25,29]^ Our reported results, although from a small sample population, also showed similar no differences between the 3 groups with regard to overall survival and disease-free survival in the elderly patients. Indeed, the 5-year oncological safety of minimally invasive laparoscopic surgery for colorectal cancers in the elderly patients has been evaluated equal to traditional open surgery in terms of overall survival, disease-free survival, and recurrence.^[30]^ These results collectively confirm the long-term oncological safety of HALS in treatment of old right colon cancers.

The results should be cautiously interpreted with existing limitations. The retrospective designed study was the naturally disadvantage. However, the groups were well balanced in terms of gender, BMI, ASA scores, preoperative blood test, and oncological stages. Secondly, univariate and multivariate analysis were abolished, due to small sample size in groups, to evaluate risk factors for postoperative complications and cancer-specific survival. Thirdly, the number of patients in HALS group was smaller than that in the LS groups. Although the feasibility of HALS in right colon cancer has been validated in general patients by our leading surgeon, the learning curve for specific elderly cancer patients may be various. A larger sample in a well-designed prospective cohort comparing the outcomes between HALS and LS is recommended.

In conclusion, compared with OS or LS, HALS may be more favorable in treatment of elderly right colon cancers with decreased surgical time and postoperative recovery, and comparable cancer-specific survivals.

## Author contributions

**Conceptualization:** Ziqiang Wang.

**Data curation:** Pingfan Ma, Wanbin He, Liang Bi.

**Visualization:** Ziqiang Wang.

**Writing – original draft:** Mingtian Wei.

**Writing – review & editing:** Xubing Zhang.
